# Post-traumatic stress disorder among COVID-19-affected high-risk cardiac patients

**DOI:** 10.1093/inthealth/ihad017

**Published:** 2023-03-13

**Authors:** Ulrich Wesemann, Ali Sahebi, Julia Vogel, Kai Köhler, Jana Kupusovic, Tienush Rassaf, Johannes Siebermair

**Affiliations:** Department of Psychiatry, Psychotherapy and Psychotraumatology, Bundeswehr Hospital, 10115, Berlin, Germany; Non-communicable Diseases Research Center, Ilam University of Medical Sciences, 69315-516, Ilam, Iran; Department of Cardiology and Vascular Medicine, West German Heart and Vascular Center Essen, University Duisburg-Essen, 45147, Essen, Germany; Department of Psychiatry, Psychotherapy and Psychotraumatology, Bundeswehr Hospital, 10115, Berlin, Germany; Department of Cardiology and Vascular Medicine, West German Heart and Vascular Center Essen, University Duisburg-Essen, 45147, Essen, Germany; Department of Cardiology and Vascular Medicine, West German Heart and Vascular Center Essen, University Duisburg-Essen, 45147, Essen, Germany; Department of Cardiology and Vascular Medicine, West German Heart and Vascular Center Essen, University Duisburg-Essen, 45147, Essen, Germany

**Keywords:** age, cardiology, COVID-19, gender, inpatients, PTSD

## Abstract

**Background:**

During the first coronavirus disease 2019 (COVID-19) wave there was a high prevalence of mental health impairments and post-traumatic stress disorder (PTSD), particularly in patients with comorbid cardiac diseases.

**Methods:**

During waves 2–5, all hospitalized patients with cardiac problems and suspected COVID-19 were eligible to participate in this study.

**Results:**

The prevalence of PTSD was 31.4% (n=48) in 153 participants. No age- and gender-related differences for PTSD were found.

**Conclusions:**

The prevalence is lower than during the first wave but higher than in patients reported in other studies who were isolated at home. Routine mental health assessments are strongly recommended for patients at risk.

## Introduction

Coronavirus disease 2019 (COVID-19) can be regarded as a severe stressor on mental health in the general population and especially of those affected.^1,2^ During the first wave of COVID-19, there was a 37.9% point prevalence of post-traumatic stress disorder (PTSD) among affected elderly at-risk patients with comorbid cardiovascular conditions.^1^ This high rate was attributed to the high mortality rate during the first wave, the lack of adequate treatment and disastrous media reports showing corpses outside overcrowded hospitals in several countries.^1^ But not only those affected were concerned. An umbrella review reported a 13.5% prevalence among healthcare workers dealing with sufferers.^3^ During the fourth wave, another study found a 22.9% point prevalence in affected younger patients isolated at home. Older age and male sex proved to be protective factors.^2^ The aim of this study was to investigate the point prevalence in at-risk patients hospitalized with suspected COVID-19 in the second to fifth COVID-19 waves and to look for age and gender differences. We hypothesized a lower prevalence rate of PTSD than in the first wave and a decline across waves. Furthermore, we expected a higher prevalence rate in our sample than in the younger non-hospitalized population without other major comorbidities.

## Methods

All patients with suspected COVID-19 infection at admission who were hospitalized between January 2021 and May 2022 due to severe cardiovascular problems, chronic lung diseases or oncological diseases at the University Hospital Essen were included in the study. The study was approved by the Ethics Committee of the University of Essen (22-10982-BO). All patients filled out several questionnaires as part of routine diagnostics. This included the Post-Traumatic Stress Disorder (PCL-5) Checklist, which measures PTSD with 20 items on a 5-point Likert scale. From 182 possible participants ≥18 y of age, data from 153 at-risk patients were obtained (inclusion rate 84.1%). The main reasons for non-participation were immobility and lack of concentration; the main diagnostic categories were coronary artery disease (n=33 [21.6%]), congestive heart failure (n=32 [20.9%]), atrial fibrillation/flutter (n=34 [22.2%]) and others (n=54 [35.3%]). Ages ranged between 19 and 92 years (mean 62 y; median 63 y; quartiles 19–52, 53–63, 64–75 and 76–92 y) were included in the study and 78 participants (51%) were female. The participants were assigned to the different COVID waves based on the date of admission. After calculating the point prevalence, 2×2 χ^2^ tests were used to look for gender and 2×4 χ^2^ tests were used for age, broken down into quartiles as well as for COVID wave 2–5 differences. In addition, a binary logistic regression analysis was performed to investigate the influence of age and gender on PTSD.

## Results

The point prevalence of PTSD in the at-risk patients was 31.4% (n=48 [95% confidence interval {CI} 23.9 to 38.9]). With χ^2^(1, N=153)=0.39; p=0.53, there was no difference in gender. Looking at age, there was also no difference: χ^2^(3, N=153)=1.47; p=0.69. With χ^2^(2, N=153)=1.80; p=0.41, the result of the binary logistic regression analysis remained without significant differences: odds ratio (OR) for male gender 1.19 (95% CI 0.60 to 2.36) and age 0.99 (95% CI 0.97 to 1.01).

Looking at COVID waves 2–5, with χ^2^(3, N=153)=3.27; p=0.35, there were no significant differences in the PTSD prevalence rates, as shown in Table 1.

**Table 1.** 2×4 χ^2^ tests for group differences between COVID-19 waves 2–5 and PTSD in hospitalized at-risk patients with additional cardiovascular diseases

COVID-19 wave	Patients without PTSD, n (expected cell totals) [χ^2^ statistic for each cell]	Patients with PTSD, n (expected cell totals) [χ^2^ statistic for each cell]	Total, n	χ^2^	p-Value
2	14 (16.47) [0.37]	10 (7.53) [0.81]	24		
3	55 (50.10) [0.48]	18 (22.90) [1.05]	73		
4	24 (26.08) [0.17]	14 (11.92) [0.36]	38		
5	12 (12.35) [0.01]	6 (5.65) [0.02]	18		
Total	105	48	153	3.27	0.352

Wave II: calendar week 40 2020–08 2021; Wave III: 09 2021–23 2021; Wave IV: 31 2021–51 2021; Wave V: 52 2021; (Expected cell totals); [chi-square statistic for each cell].

## Discussion

The results suggesting that patients affected by COVID-19 during waves 2–5 experienced less psychological distress compared with patients during the first wave are consistent with previous studies (e.g. Nguyen et al.^2^ and Serra et al.^4^). This is attributed to risk perception. Mortality rates decreased significantly over time, the locus of control was strengthened by vaccines and there was less media attention due to an habituation effect. However, we found no further decrease in PTSD rates across waves 2–5. This could be because our sample size is too small or the risk assessment is different in this very vulnerable group.

In contrast to Nguyen et al.,^2^ we did not find gender or age as risk or resilience factors for PTSD. With a 31.4% prevalence rate, our results also are remarkably higher than the 22.9% reported by Nguyen et al.^2^ We think this is due to several factors. First, our population had been hospitalized, reducing protective factors such as reported social support. Second, our patients had additional life-threatening comorbidities that led to poorer prognosis. And third, these comorbidities alone could have been the cause of PTSD. However, both studies are limited by the use of questionnaires, which often overestimate prevalence due to specificity.^5^ Further research is needed to examine age and gender differences in different groups of affected persons with regard to comorbidities and vaccination status.

## Conclusions

Routine mental health assessments and psychological counselling are strongly recommended for high-risk patients. Positive effects are expected not only for the psychological stress, but also for the purely physical complaints. However, these effects must be examined in further studies. This could further optimize treatment and prepare them for their next health challenges.

## Data Availability

The datasets generated during the current study are available from the corresponding author upon reasonable request.
